# Supplementary material for: Mucin-induced surface dispersal of Staphylococcus aureus and Staphylococcus epidermidis via quorum-sensing dependent and independent mechanisms
Source: mBio. 2024 Jul 2;15(8):e01562-24. doi: 10.1128/mbio.01562-24 (PMC11323471; doi:10.1128/mbio.01562-24)
Supplement: Supplemental Material — Figures S1–S8. [file mbio.01562-24-s0001.pdf]

Supplementary Information

for

Mucin-induced surface dispersal of *Staphylococcus aureus* and *Staphylococcus epidermidis* via quorum sensing-dependent and independent mechanisms

Kristin M. Jacob, K.M., Santiago Hernandez-Villamizar, Neal D. Hammer, and Gemma Reguera*

Department of Microbiology, Genetics & Immunology, Michigan State University, East Lansing, MI 48824

***Corresponding author:**

567 Wilson Rd, Rm. 6190

Biomedical and Physical Science building

Michigan State University

East Lansing, MI 48824

Keywords: staphylococcus, mucin, mucosa, motility, dendritic formation, quorum sensing

Supplementary Figures

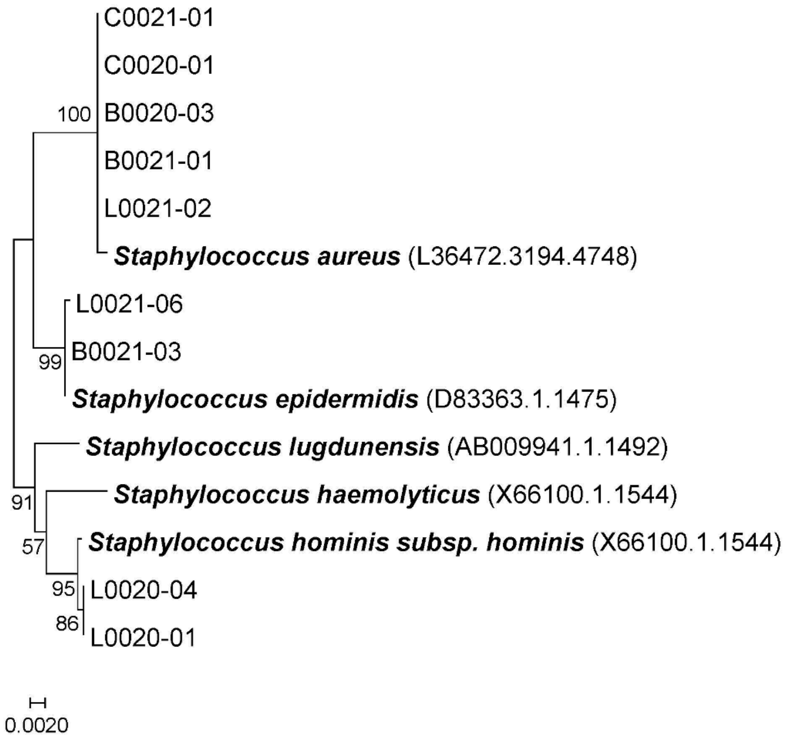

FIG S1: 16S rRNA phylogenetic tree of perioral staphylococcal strains used in this study. Maximum likelihood tree constructed with the full-length 16S rRNA gene from staphylococci isolated from otic (L), buccal (B) or oropharyngeal (C) secretions and their closest relative reference sequences (accession numbers). Scale bar indicates 2% sequence divergence filtered to a conservation threshold above 79% using the Living Tree Database (1, 2). Bootstrap probabilities by 1000 replicates at or above 50% are denoted by numbers at each node.

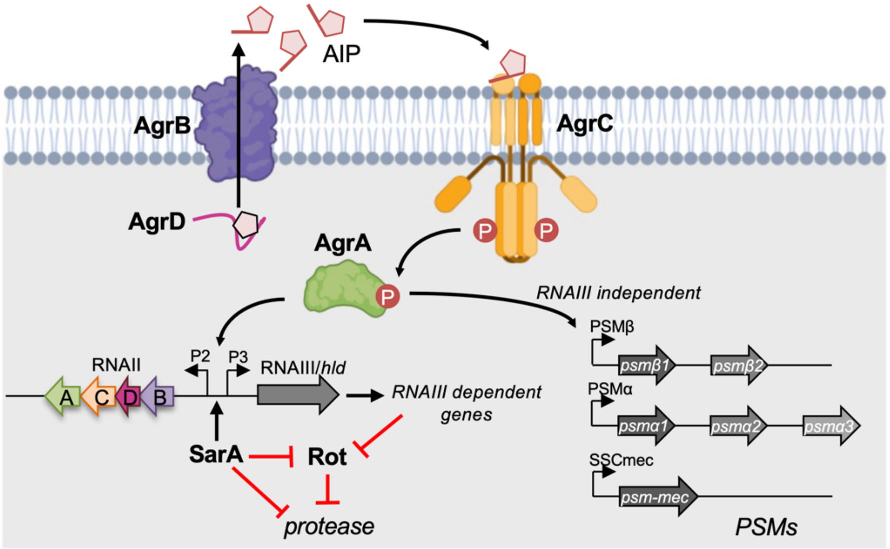

FIG S2: *S. aureus* quorum sensing regulation. Illustration of the main components of the *S. aureus* quorum sensing network, including the ArgB protease that processes the AgrD-autoinducer peptide (AIP) prior to its secretion, the AgrC histidine kinase, the AgrA response regulator, the SarA enhancer, and the Rot regulator. The AgrA-regulated operons are also shown, including those encoding the phenol-soluble modulins (PSMs).

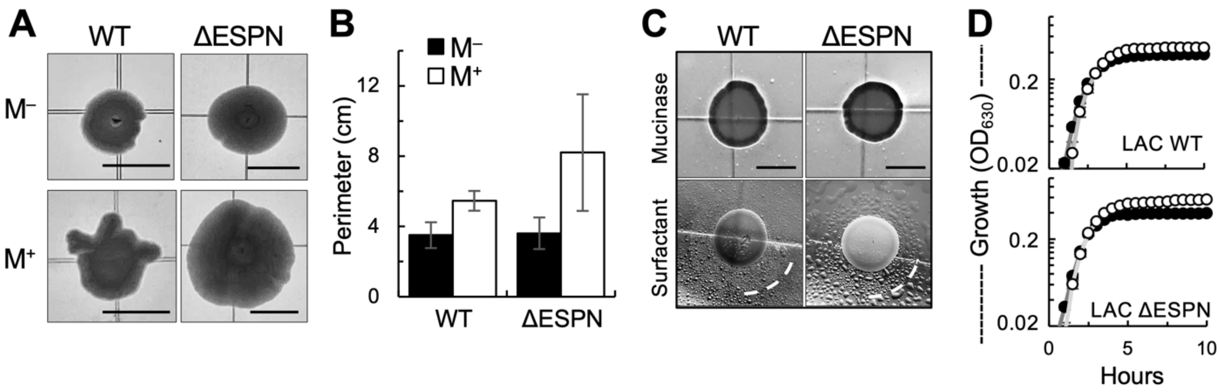

FIG. S3: Effect of mucin on surface dispersal and growth of *S. aureus* LAC and a protease-defective mutant (ΔESPN). (**A-B**) Representative morphotypes (**A**) and perimeters (average and standard deviation of at least 4 replicates) of 42-h colonies on plates without (M^-) or with (M^+) porcine stomach mucin (0.4% w/v). (**C-D**) Mucinase activity (haloes of mucin degradation around colonies) and surfactant activity (haloes of oil drop dispersion with the atomized oil assay, as marked with a dashed line) of the mucin-grown colonies (**C**). Planktonic growth in 50% TSB with (open symbols) or without (closed circles) mucin supplementation (**D**) (average and standard deviation of 8 replicated microtiter wells per condition).

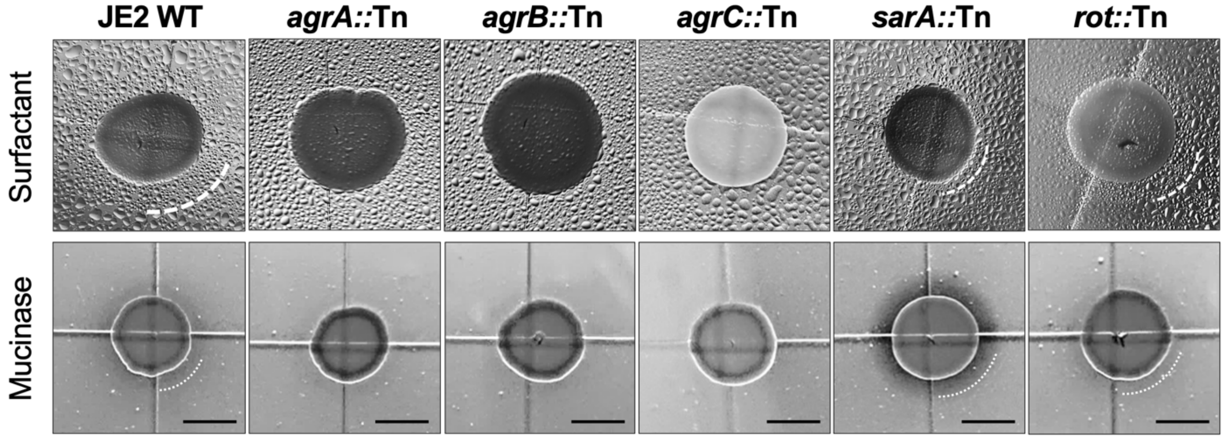

FIG. S4: Effect of mucin on PSM and mucinase production by *S. aureus* and mutants. The figure shows representative colony phenotypes on 0.3% (w/v) motility TSA plates with porcine stomach mucin (0.4% w/v) after airbrushing with a fine mist of mineral oil (atomized oil assay, surfactant-active halo indicative of PSM production) and on 1.5% TSA plates with mucin to visualize haloes of mucin degradation (mucinase activity).

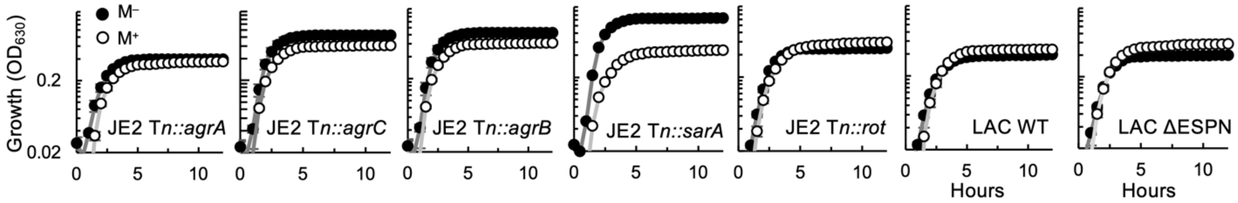

FIG S5: Mucin growth. Planktonic growth of *S. aureus* JE2 and LAC strains (WT and mutants) in 50% tryptic soy broth (TSB) in the presence (M^+) or absence ($M^–$) of commercial porcine stomach mucin.

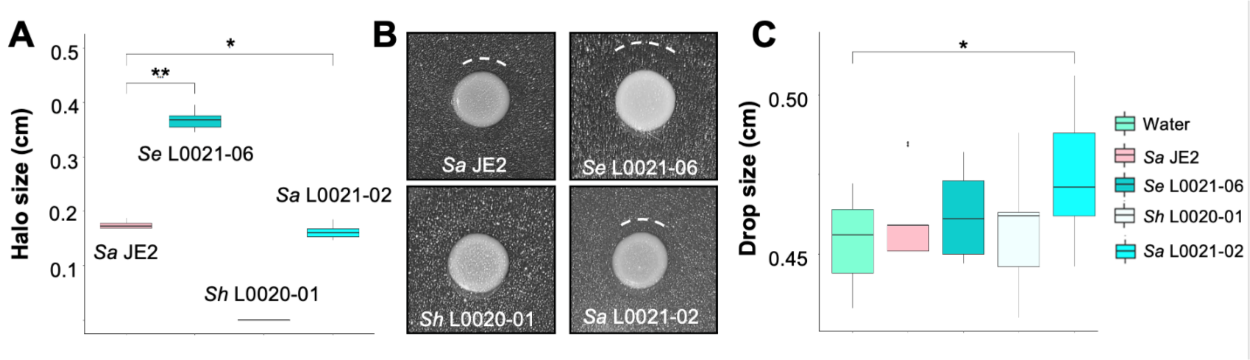

Fig. S6: PSM surfactant activity of *S. aureus* JE2 and perioral isolates of *S. aureus* (*Sa*), *S. epidermidis* (*Se*) and *S. hominis* (*Sh*). (A-B) Size (average and standard deviation of triplicate colonies) of the surfactant-active zone around colonies grown on 1.5% TSA plates (**A**) based on the size of the halo of oil dispersion visualized around colonies after airbrushing a fine mist of mineral oil (panel **B** shows representative images of colonies with the edge of the halo, when present, highlighted with a dashed white line). (**C**) Drop expansion of PSM colony extracts on Parafilm M (average and standard deviation of triplicate PSMs extracts for each strain).

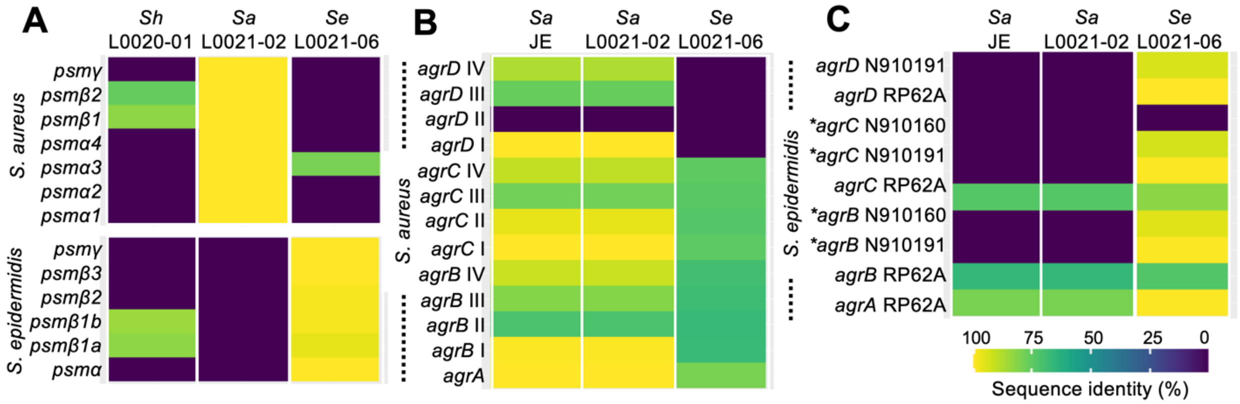

Fig. S7: Heatmap of *agr* and *psm* sequence identity between test and reference strains. The reference genomes (GenBank accession number in parenthesis)used for sequence analyses were *S. aureus* RN4282 (*agr* I: accession number U85097), SA502A (*agr* II: accession number AF001782), RN8462 (*agr* III: accession number AF001783) and *agr* locus (*agr* IV: accession number AF288215) for *agr* genes, and USA300_FPR3757 for *psm* genes (accession number: NC_007793). For *S. epidermidis*, the reference genomes utilized were RP62A (accession number: CP000029), N910191M (accession number: AF346725), and N910160 (accession number: AF346724) for *agr* genes, and RP62A (accession number: CP000029) and ATCC 14990 (accession number: CP035288) for *psm* genes (3). The test strains were *S. aureus* JE2 (CP020619) and three perioral isolates (*S. hominis* L0020-01, *S. aureus* L0021-02 and *S. epidermidis* L0021-06) whose genomes were sequenced and assembled for this study. The sequence identity (%) between the *psm* (**A**) and *agr* (**B-C**) genes in the reference genomes (*S. aureus* or *S. epidermidis*) and top matches in the test strains are shown. Partial genes are shown with an asterisk (*)

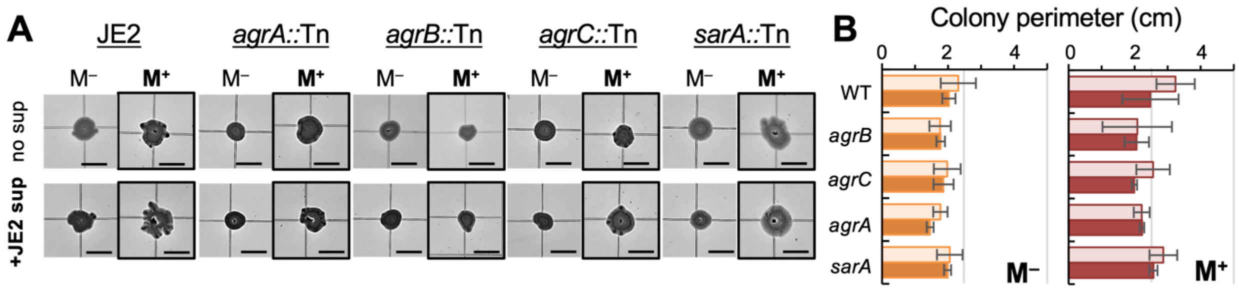

Fig. S8: Effect of JE2 supernatants on colony expansion of the JE2 and quorum-sensing mutants. (**A**) Colony morphotypes on 0.3% (w/v) TSA with (M^+) or without ($M^–$) porcine stomach mucin supplementation (0.4% w/v) after 18 h of incubation at 37°C. Colonies treated with supernatant fluids from *S. aureus* JE2 stationary phase cultures are shown in reference to untreated controls. (**B**) Perimeter (average and standard deviation of triplicate colonies) of 18-h colonies in the presence (M^+) or absence ($M^–$) of 0.4% porcine stomach mucin, with (dark shade) or without (light shade) supernatant additions.

Supplementary references

1. Yarza P, Richter M, Peplies J, Euzeby J, Amann R, Schleifer KH, Ludwig W, Glockner FO, Rossello-Mora R. 2008. The All-Species Living Tree project: A 16S rRNA-based phylogenetic tree of all sequenced type strains. Syst Appl Microbiol 31:241-250. doi:10.1016/j.syapm.2008.07.001.
2. Yilmaz P, Parfrey LW, Yarza P, Gerken J, Pruesse E, Quast C, Schweer T, Peplies J, Ludwig W, Glockner FO. 2014. The SILVA and "All-species Living Tree Project (LTP)" taxonomic frameworks. Nucl Acids Res 42:D643-D648. doi:10.1093/nar/gkt1209.
3. Cheung GY, Joo HS, Chatterjee SS, Otto M. 2014. Phenol-soluble modulins--critical determinants of staphylococcal virulence. FEMS Microbiol Rev 38:698-719. doi:10.1111/1574-6976.12057.
